# Influence of Particle Size and Xylanase Pretreatment of Proso Millet Bran on Physical, Sensory and Nutritive Features of Gluten-Free Bread

**DOI:** 10.17113/ftb.61.01.23.7776

**Published:** 2023-03

**Authors:** Dubravka Novotni, Ljiljana Nanjara, Lucija Štrkalj, Saša Drakula, Nikolina Čukelj Mustač, Bojana Voučko, Duška Ćurić

**Affiliations:** 1 University of Zagreb, Faculty of Food Technology and Biotechnology, Pierottijeva 6, 10000 Zagreb, Croatia; 2 University of Applied Sciences ‘Marko Marulić’, Petra Krešimira IV 30, 22300 Knin, Croatia; 3 Division of Food Sciences, Nutrition and Dietetics, School of Biosciences, University of Nottingham, Sutton Bonington Campus, Loughborough, LE12 5RD, UK

**Keywords:** cereal by-product, cryogenic milling, dietary fibre, mineral bioaccessibility, phenolic acids

## Abstract

**Research background:**

Millet bran is a by-product rich in dietary fibre, micronutrients and bioactive compounds which are often deficient in a gluten-free diet. Previously, cryogenic grinding has been shown to improve the functionality of bran to some extent, although it offered limited benefits for bread making. This study aims to investigate the effects of adding proso millet bran depending on its particle size and xylanase pretreatment on the physicochemical, sensory and nutritional properties of gluten-free pan bread.

**Experimental approach:**

Coarse bran (*d*_50_=223 μm) was ground to medium size (*d*_50_=157 μm) using an ultracentrifugal mill or to superfine particles (*d*_50_=8 μm) using a cryomill. Millet bran presoaked in water (for 16 h at 55 °C) with or without the addition of fungal xylanase (10 U/g) replaced 10% of the rice flour in the control bread. Bread specific volume, crumb texture, colour and viscosity were measured instrumentally. Along with proximate composition, the content of soluble and insoluble fibre, total phenolic compounds (TPC) and phenolic acids as well as total and bioaccessible minerals of bread were assessed. Sensory analysis of the bread samples included a descriptive, hedonic and ranking test.

**Results and conclusions:**

Dietary fibre content (7.3-8.6 g/100 g) and TPC (42-57 mg/100 g) on dry mass basis of the bread loaves depended on bran particle size and xylanase pretreatment. The effect of xylanase pretreatment was most evident in the loaves with medium bran size in terms of higher content of fibre soluble in ethanol (45%) and free ferulic acid content (5%), improved bread volume (6%), crumb softness (16%) and elasticity (7%), but lower chewiness (15%) and viscosity (20-32%). Bread bitterness and dark colour were increased after adding medium-sized bran but its bitter aftertaste, crust crookedness, crumb hardness and graininess were reduced with xylanase pretreatment. Although bran addition impaired protein digestibility, it enriched the bread with iron (341%), magnesium (74%), copper (56%) and zinc (7.5%). Xylanase pretreatment of the bran resulted in the improved bioaccessibility of zinc and copper of the enriched bread compared to the control and bread without xylanase.

**Novelty and scientific contribution:**

Application of xylanase to medium sized bran obtained by ultracentrifugal grinding was more successful than its application to superfine bran obtained by the multistage cryogrinding as it resulted in more soluble fibre in gluten-free bread. Moreover, xylanase was proven beneficial in maintaining desirable bread sensory properties and mineral bioaccessibility.

## INTRODUCTION

The actual prevalence of wheat- or gluten-related disorders is not known (*1*), but it has been estimated that approx. 7% of the world’s population must follow wheat- or gluten-free diet (*2*). A gluten-free diet is often associated with nutritional deficiencies (*3*). For example, gluten-free bread often contains high amounts of rapidly digestible carbohydrates and fat but low amounts of dietary fibre, micronutrients and bioactive compounds. Therefore, the enrichment of gluten-free bread is constantly being researched, with natural ingredients being particularly valued (*4*).

Millet bran is a by-product of grain decortication that can be used to enrich gluten-free bread with dietary fibre and phenolic compounds, and even improve its volume and crumb softness (*5*). Millet bran fibre consists of insoluble arabinoxylans, lignin, cellulose, cutin and silicium dioxide (*6*-*8*), as well as phenolic acids that are mostly bound to arabinoxylans of the cell wall (*5*). Although they have beneficial effects on health, fibre and phenolics may also impair protein digestibility (*9*, *10*). In addition, dietary fibre can bind minerals and hinder their bioavailability (*11*). The bioavailability of nutrients can be improved by the bread making process, but only to a certain extent. Therefore, to maximize the functional potential of bran, new processing techniques are being explored (*4*).

Ultrafine grinding decomposes grain by-products at the subcellular level, improving their functional properties, nutritional value and nutrient bioavailability (*12*-*14*). It has already been shown that the particle size of bran used in bread making generally affects the technological, sensory and nutritional quality of bread (*5*, *15*, *16*). Grinding cereal bran to superfine and ultrafine sizes improves the ratio of soluble to insoluble fibre and promotes the release of bioactive compounds such as phenolic compounds from the fibre matrix (*14*, *17*, *18*). Although micronisation of millet bran to a particle size of 26–46 μm slightly increases the antioxidant activity as well as soluble fibre content, the effect of grinding after adding the bran to gluten-free bread is insignificant (*5*). Therefore, other methods of processing bran for bread making need to be investigated to extend the limited benefits of grinding.

Xylanases are used as baking aids to improve bread volume, shape, crumb structure and texture, shelf-life and nutrient bioavailability (*8*, *19*, *20*). These hydrolytic enzymes convert water-insoluble arabinoxylans into soluble forms by randomly cleaving the β-1,4 backbone of the arabinoxylans (*21*). Nevertheless, the influence of millet bran particle size on the success of xylanase pretreatment and its combined effect on the quality features of the resulting gluten-free bread have not yet been investigated.

In continuation of our previous study (*5*), in this work we aim to investigate the interaction between the particle size (coarse, medium and superfine) of proso millet bran and xylanase pretreatment on the physicochemical properties of the gluten-free bread, as well as on the soluble fibre, phenolic acids and total phenolic content. The proximate composition and nutrient profile (*i.e*. mineral bioaccessibility, *in vitro* protein digestibility) and sensory attributes of the selected enriched bread were compared with a control rice bread.

## MATERIALS AND METHODS

### Materials and chemicals

The refined rice flour containing 12.7% moisture, 9% proteins and 0.33% ash was donated by Naše Klasje (Zagreb, Croatia). The bran of proso millet (*Panicum miliaceum,* variety Sonček, crop year 2019) obtained after grain decortication and sieving was a gift from an industrial mill (Mlinopek, Murska Sobota, Slovenia). The bran contained (in %): moisture 10.8, proteins 10.1, fat 6.7, ash 4.5 and (in mg/100 g): magnesium 212, iron 6.4, zinc 1.4 and copper 0.90 (determined as described below for bread). The endoxylanase activity of the bran was 0.045 U/g, as determined by the Xylazyme AX Tablet assay (Megazyme, Bray, Ireland) using *Asperigillus niger* control xylanase. Fungal xylanase from *Trichoderma longibrachiatum* with the activity of 1000 U/g up to 83 *°*C was kindly provided by Bio-Cat (Richmond, VA, USA). Other ingredients used in bread making were: instant yeast (Dr Oetker, Zagreb, Croatia), sugar (Viro, Virovitica, Croatia), salt (Solana Pag, Pag, Croatia) and butter (Dukat, Zagreb, Croatia).

Proximate composition, dietary fibre, total phenolic and phenolic acid content of the samples were analysed using commercially procured chemicals and standards: methanol (J.T. Baker, Phillipsburg, NJ, USA), ethanol p.a., petrol ether, sulphuric and nitric acid (Carlo Erba, Cornaredo, Italy), ethanol denaturated 96% (Kemika, Zagreb, Croatia), nitric acid, hydrochloric acid (37%), and acetonitrile (65%, Carlo Erba, Val-de-Reuil, France), sodium hydrogencarbonate and d(-)-fructose ≥99.5% (Acros Organics, Geel, Belgium), acetone, boric acid, sodium hydroxide and d(+)-glucose p.a. (Gram-Mol, Zagreb, Croatia), Kjeldahl tablets (Merck, Darmstadt, Germany), lanthanum(III) chloride heptahydrate ≥98% (BDH Prolabo, VWR International Ltd, Lutterworth, UK), ferulic acid (Fluka, Buchs, Switzerland), saccharose (99.5%), vanillic acid, gallic acid, 4-hydroxybenzoic acid, *p*-coumaric acid, 3,5-dichloro-4-hydroxybenzoic acid and Folin-Ciocalteu reagent (all Sigma-Aldrich, Merck, Darmstadt, Germany). For the determination of mineral content and bioacessibility standards of copper, iron, zinc and magnesium (1 mg/mL; Supelco, Darmstadt, Germany), pepsin (crystalline, 3300 U/mg), pancreatin from porcine pancreas (activity equivalent to 4×USP) and porcine bile extract (all Sigma-Aldrich, Merck) were used.

### Bran preparation

The particle size of the millet bran obtained from the industrial mill was described as coarse. It was further ground to medium particle size in an ultra-centrifugal mill (ZM 200; Retsch, Haan, Germany) with a ring sieve of aperture size of 200 µm, or to superfine particle size in a cryogenic ball mill (CryoMill, Retsch). Cryogrinding was carried out with 8 g of sample in a 50-mL stainless steel container with a steel ball (25 mm diameter) and a vibration frequency of 30 Hz for 8 min under nitrogen cooling. It was performed three times in succession, with sieving in between at aperture size of 55 µm. The particle size distribution of the bran was determined by the laser diffraction according to AACC method 55-40.01 (*22*) using the Mastersizer 2000 apparatus with Scirocco 2000 dry dispersion unit (Malvern Instruments, Malvern, UK). The particle size distribution was calculated using a refractive index of 1.5 and the obscuration 3% (*5*) and is shown in Table 1.

**Table 1:** Particle size (*d*) distribution of rice flour and millet bran samples

Sample	*d*_50_/μm		*d*_90_/μm	Span/μm	*d*_32_/μm	*S*/(m^2^/kg)
Rice flour		(176.0±0.7)^b^	(374.5±0.6)^c^	(1.8±0.05)^c^	(86.6±0.9)^b^	(28.3±1.1)^c^
Coarse bran		(223.4±4.3)^a^	(422.2±2.6)^a^	(1.6±0.04)^d^	(112.9±4.8)^a^	(21.7±0.9)^d^
Medium bran		(157.0±0.2)^c^	(381.3±0.2)^b^	(2.3±0.03)^b^	(53.2±0.4)^c^	(46.1±0.4)^b^
Superfine bran		(7.9±0.1)^d^	(31.8±0.3)^d^	(3.7±0.04)^a^	(5.45±0.1)^d^	(449.0±10.7)^a^

The results are expressed as mean value±standard deviation, *N*=3. Mean values with different letters in superscript within the same column differ significantly at p<0.05. *d*_32_=surface weighted or Sauter mean diameter, *S*=specific surface area, span=width of distribution

Prior to baking, the bran was soaked in distilled water (1:2.5 g/mL) with or without fungal xylanase at an activity of 10 U/g for 16 h at 55 °C, pH=5.6, in a shaking water bath (Stuart SBS40; Bibby Scientific Ltd., Stone, UK) (*5*).

### Bread preparation

Control bread without the bran or xylanase was prepared according to a previously described recipe (*5*) consisting of rice flour (400 g), water (352 mL), sugar (16.7 g), instant yeast (5.7 g), salt (5.3 g) and butter (4.2 g). In the first phase, six bread samples were baked with bran (39.15 g, *i.e.* 10% of the rice flour, corrected for moisture content) differing in particle size (coarse, medium or superfine), pretreated with or without xylanase, and with the amount of water indicated in Table 2, according to the same recipe as the control bread. In the second phase, the selected bread samples enriched with millet bran were compared with the control rice bread for their nutritive and sensory features. Twelve samples (110 g) of bread from each recipe were baked in pans in two separate batches as described previously (*5*).

**Table 2:** Xylanase and water addition in recipes and analysis of bread content of moisture, dietary fibre, total phenolic content (TPC) and phenolic acids on dry mass basis depending on the bran particle size (coarse, medium or superfine) and xylanase (X) addition

**Parameter**	Coarse	Coarse+X	Medium	Medium+X	Superfine	Superfine+X
**Xylanase/(U/g)**	0	10	0	10	0	10
***V*(water)/mL**	449	449	449	449	427	427
***w*(moisture)/(g/100 g)**	(47.0±0.1)^a^	(46.75±0.08)^ab^	(46.78±0.04)^ab^	(46.4±0.1)^b^	(43.65±0.09)^c^	(43.5±0.1)^c^
***w*(TDF)/(g/100 g)**	(8.27±0.01)^ab^	(8.33±0.05)^ab^	(8.3±0.2)^b^	(8.63±0.01)^a^	(7.75±0.02)^c^	(7.30±0.07)^d^
***w*(IDF)/(g/100 g)**	(6.7±0.1)^a^	(6.8±0.1)^a^	(6.4±0.2)	(6.47±0.06)^ab^	(6.3±0.1)^bc^	(5.98±0.07)^c^
***w*(SDFP)/(g/100 g)**	(0.6±0.1)^ab^	(0.49±0.03)^ab^	(0.75±0.09)^a^	(0.80±0.05)^a^	(0.31±0.10)^bc^	(0.14±0.02)^c^
***w*(SDFS)/(g/100 g)**	(0.95±0.02)^d^	(1.03±0.03)^c^	(0.90±0.01)^d^	(1.36±0.01)^a^	(1.16±0.01)^b^	(1.18±0.01)^b^
***w*(TPC as GAE)/(mg/100 g)**	(50.9±2.2)^ab^	(46.0±0.2)^bc^	(42.0±0.9)^c^	(49.1±1.8)^b^	(56.9±1.2)^a^	(45.9±2.1)^bc^
***w*(total phenolic acid)/(mg/100 g)**	(4.40±0.04)^a^	(4.4±0.4)^a^	(4.2±0.2)^a^	(4.4±0.3)^a^	(4.4±0.23^a^	(4.5±0.4)^a^
***w*(ferulic acid)/(mg/100 g)**	(1.61±0.02)^b^	(1.89±0.04)^a^	(1.72±0.04)^ab^	(1.80±0.08)^a^	(1.59±0.07)^b^	(1.84±0.08)^a^
***w*(gallic acid)/(mg/100 g)**	(0.777±0.006)^cd^	(0.892±0.005)^c^	(0.72±0.04)^d^	(1.146±0.002)^ab^	(1.19±0.06)^a^	(1.04±0.06)^b^
***w*(4-hydroxybenzoic acid)/(mg/100 g)**	(1.04±0.03)^a^	(1.007±0.007)^a^	(0.88±0.04)^ab^	(0.81±0.06)^b^	(0.72±0.08)^b^	(0.74±0.03)^b^
***w*(vanillic acid)/(mg/100 g)**	(0.71±0.02)^a^	(0.63±0.05)^ab^	(0.57±0.06)^b^	(0.52±0.05)^b^	(0.58±0.02)^b^	(0.55±0.05)^b^
***w*(*p*-coumaric acid)/(mg/100 g)**	(0.26±0.01)^b^	(0.28±0.02)^ab^	(0.28±0.01)^ab^	(0.26±0.04)^b^	(0.31±0.02)^ab^	(0.34±0.03)^a^

The results are expressed as mean value±standard deviation, *N*≥2. TDF=total dietary fibre, IDF=insoluble dietary fibre, SDFP=fibre soluble in water but precipitated in 78% aqueous ethanol, SDFS=fibre soluble in water but not precipitated in 78% aqueous ethanol. GAE=gallic acid equivalents. Mean values with different letters in superscript within the same row are significantly different at p<0.05

### Determination of bread physical properties

The physical properties of the bread samples were measured in six replicates. The volume of the weighed samples was measured by rapeseed displacement according to the AACC method 10-05-01 (*22*). Bread specific volume was calculated as the volume to mass ratio measured 1 h after baking. The crumb texture profile was measured with a TA.HDplus Texture Analyser (Stable Micro Systems, Godalming, UK) using a 25 mm probe with a test speed of 2 mm/s, 50% strain and 30 s pause. Crumb colour parameters were evaluated using a colourimeter (spectrophotometer CM-3500 D; Konica Minolta, Milton Keynes, UK) in the CIELab system. The lightness L* defines black at 0 and white at 100. The a* indicates greenness if values are negative and redness with positive values, while the b* negative numbers indicate the blueness while positive values show the intensity of yellow.

The viscosity of bread suspension in water was determined in duplicate according to AACC method 61-02.01 (*22*), using a microviscoamylograph (Brabender, Duisburg, Germany), in the measuring range 250 cmg. The viscosity (expressed in Brabender units, BU) of bread crumb (15 g) suspended in water (105 mL) was recorded during heating to 92 °C at 7.5 °C/min, holding the temperature (92 °C) for 1 min, and cooling to 50 °C, with constant stirring.

### Determination of bread proximate composition and nutritive value

Protein (nitrogen×6.25) (AACC method 46-12.01), moisture (AACC method 44-15.02), ash and total fat (both AACC method 30-10.01) contents were determined in duplicate according to AACC methods (*22*). The obtained values were subtracted from the total mass and the difference was considered as carbohydrates. The energy value was calculated by multiplying the carbohydrate and protein content by 16 kJ or 4 kcal, fat content by 36 kJ or 9 kcal, and fibre content by 8 kJ or 2 kcal. Protein digestibility was determined *in vitro* using the K-PDCAAS 12/19 Megazyme kit.

Insoluble dietary fibre (IDF), fibre soluble in water but precipitated in 78% aqueous ethanol (SDFP), and fibre soluble in water and in the presence of 78% aqueous ethanol (SDFS) were determined according to AOAC method 2011.25 (*23*) using the K-INTDF enzyme kit (Megazyme). SDFS were analysed using an HPLC system (Shimadzu, Kyoto, Japan) with the MetaCarb 67C column (Agilent Technologies, Santa Clara, CA, USA).

The same HPLC system and refractive index detector with the MetaCarb 67H column (300 mm×6.5 mm, Agilent) were used for the determination of sugars (sucrose, glucose and fructose) according to Lefebvre *et al*. (*24*). For this purpose, the supernatant was filtered through a nylon syringe filter (pore size 0.45 µm; FilterBio, Nantong, PR China) and 20 µL of the sample were injected onto the column at 40 °C. Analysis was performed by isocratic elution of the mobile phase (0.5 mM aqueous sulphuric acid solution) at a rate of 0.5 mL/min. An external standard method was used to quantify the sugars. The results are reported as the sum of the determined sugars.

Phytate content was determined spectrophotometrically using the K-PHYT 07/11 Megazyme assay kit according to the manufacturer’s instructions.

Dry ashing was performed in a muffle furnace (Heraues, Hanau, Germany) at 550 °C according to AOAC method 923.03 (*23*). The ash was dissolved in 5 mL of 5 M nitric acid and diluted to 50 mL with deionised water. For the determination of mineral bioaccessibility, samples were prepared according to the modified method of Luten *et al.* (*25*). A spectra/Por4 membrane (32 mm, 12-14 kDa molecular mass cut-off; Behr, Germany) was used for dialysis. Total and bioaccessible mineral content were measured in five replicates using a flame atomic absorption spectrometer (Perkin Elmer 2380; Norwalk, CT, USA) at *λ*=324.7 for Cu, 248.3 for Fe, 213.9 for Zn and 285.2 nm for Mg, with the remaining conditions following the manufacturer’s recommendations. In the case of magnesium determination, 1% lanthanum(III) chloride was added to the sample solution.

After ethanol extraction of free phenolics, total phenolic content (TPC) and phenolic acids were determined in triplicate as described by Čukelj Mustač *et al*. (*5*). TPC was determined spectrophotometrically using the Folin-Ciocalteu assay and expressed in mg gallic acid equivalent (GAE) per 100 g dry matter of bread (*5*). All spectrophotometric analyses were performed using a Specord 50 Plus spectrophotometer (Analytik Jena, Jena, Germany).

Individual phenolic acids were determined by HPLC (Agilent 1200 series with G1315D PDA detector; Agilent Technologies) and Kinetex 2.6 µm C18, 100 Å, 150 mm×4.60 mm (Phenomenex, Torrance, CA, USA) column (*5*). Total phenolic acids were expressed as the sum of all analysed and detected phenolic acids.

### Sensory evaluation

Sensory analysis of the bread samples included a quantitative descriptive, a hedonic and a ranking test according to ISO 6658:2017 (*26*) and ISO 8589:2007 (*27*). A half of each sample, consisting of crust and crumb, was labelled with 3-digit random numbers, and simultaneously presented to 16 previously trained panellists (14 females, 2 males, aged 23 to 56 years), all employees of the University of Zagreb, Faculty of Food Technology and Biotechnology, Zagreb, Croatia. The descriptive test included bread appearance (crust crookedness, crust and crumb colour, uniformity of crumb cell distribution), odour (raw dough, cooked rice, wet cereals, fresh bread), flavour (sweet, salty, sour, bitter, raw dough, rice, cereals, fresh bread, dusty/musty, bitter aftertaste) and texture (crumbliness after triple passing with a finger over the crumb surface in the same direction, moistness in the mouth, hardness – a force required to bite through the sample with the front teeth, chewiness – a force to prepare a sample for swallowing, adhesiveness – a force required to remove the sample from the palate, teeth and tongue, graininess – feel of particle size and shape after swallowing). The intensity of each attribute was scored from 0 (not perceived) to 10 (very intense). Overall liking was rated on a 9-point hedonic scale (1=extremely dislike, 9=extremely like) according to Svensson (*28*). In addition, the panellists ranked the bread samples according to their degree of liking from 1 (most preferred) to 3 (least preferred).

### Data analyses

Results on physical and nutritional properties of the bread samples were subjected to factorial analysis of variance (ANOVA) with *post-hoc* Tukey’s test and principal component analysis (PCA) to identify the differences between the samples as a function of bran particle size and xylanase pretreatment. Sensory analysis data were subjected to ANOVA to test for statistically significant differences among panellists and samples. Friedman’s ANOVA and Kendall concordance were used to compare the ranking of the samples. All tests were performed at the significance level p<0.05 using Statistica v. 12 software (*29*).

## RESULTS AND DISCUSSION

### Particle size distribution of flour and bran

Rice flour, and coarse and medium particle sized millet bran showed a symmetrical unimodal distribution. Medium sized bran showed a particle size distribution most similar to that of rice flour. On the other hand, the superfine bran sample exhibited a bimodal distribution (results not shown). Compared to the other samples, the cryoground bran exhibited a 20- to 28-fold the smallest mean particle size, lowest surface weighted mean (*d*_32_), but the highest specific surface area (*S*) (Table 1). Three cryogrinding steps resulted in smaller particles than ultracentrifugal grinding because the brittleness increases at sub-zero temperatures (*12*). Particle reduction is known to increase the specific surface area of a material (*30*), as well as change the structure and surface properties of the bran, which in turn alters the functional properties (*17*).

### Influence of bran particle size and xylanase on fibre and bioactive compounds of bread

Among fibre types, insoluble dietary fibre (IDF) predominated in all bread samples (Table 2), which is consistent with previous studies on millet bran (*6*, *7*, *31*). The particle size of the bran affected the amount of different fibre fractions (Table 3); it was positively correlated with total dietary fibre (TDF, R=0.81), IDF (R=0.92) and SDFP (R=0.71). Thus, pieces of bread made with superfine bran had the lowest IDF and SDFP mass fraction, but the highest SDFS mass fraction. In a previous study (*5*), there was no statistical difference in the soluble fibre mass fraction between the bread samples containing the finely cryoground and the coarse, unground millet bran. The contrasting results of this study are most likely due to the differences in the cryogrinding process, which was repeated three times here and included sieving. Thus, longer grinding time resulted in smaller particle size and higher SDFS mass fraction. Nevertheless, the lower mass fraction of TDF in the superfine bran than in the other samples indicated certain fibre loss during cryogrinding. Furthermore, xylanase pretreatment enhanced SDFS mass fraction, with the greatest effect in medium-sized bran. Thus, the ratio of soluble to insoluble fibre in the bread made with medium-sized bran pretreated with xylanase was 0.33, compared to the other two samples with an average ratio of 0.23. There are two possible reasons for the obtained results. One reason could be the larger specific surface area of the ground bran, which greatly enhances enzyme adsorption (*13*), than of coarse bran, which is resistant to enzyme hydrolysis because of its large particle size (*32*). On the other hand, a possible explanation for the lack of enzymatic action on superfine bran could be the release of otherwise fibre-bound xylanase inhibitors after cryogrinding (*33*, *34*).

**Table 3:** The p-values of factorial analysis of variance of bran particle sizes (coarse, medium or fine) and the presence of xylanase for pretreatment as well as their interaction influencing bread properties

Parameter	Bran particle size	Xylanase addition	Particle size×xylanase
Insoluble fibre	0.001	0.395	0.084
SDFP	<0.001	0.173	0.213
SDFS	<0.001	<0.001	<0.001
TPC	0.005	0.018	<0.001
Ferulic acid	0.956	0.015	0.121
Gallic acid	<0.001	<0.001	<0.001
4-hydroxybenzoic acid	<0.001	0.347	0.528
Vanillic acid	0.001	0.031	0.841
*p*-Coumaric acid	0.007	0.553	0.272
Specific volume	<0.001	0.686	0.002
Lightness L*	<0.001	<0.001	<0.001
Redness a*	<0.001	0.077	0.012
Yellowness b*	<0.001	<0.001	<0.001
Crumb hardness	<0.001	<0.001	<0.001
Resilience	<0.001	0.806	0.002
Cohesiveness	<0.001	0.883	0.002
Chewiness	0.001	<0.001	0.008
Peak viscosity	0.089	<0.001	0.001
Cold paste viscosity	0.028	<0.001	<0.001
Setback viscosity	0.013	<0.001	<0.001

SDFP=fibre soluble in water but precipitated in 78% aqueous ethanol, SDFS=fibre soluble in water but not precipitated in 78% aqueous ethanol, TPC=total phenolic content

Furthermore, TPC depended significantly on the interaction between the particle size and the xylanase pretreatment (Table 3). Bread loaves from superfine bran without xylanase had the highest mass fraction of TPC, which could be a consequence of the release of phenolics by cryogrinding, as previously reported (*14*, *18*, *30*). In contrast to cryogrinding, ultracentrifugal grinding increases the temperature of the sample, which could lead to the deterioration of phenolics. The positive influence of xylanase pretreatment on the TPC was found only in the bread made with medium-sized bran, which could be related to fibre solubilisation, while it had a reducing effect in the bread made with superfine bran.

Ferulic acid was the most abundant phenolic acid in the loaves of bread manufactured within this study, as it is the amplest phenolic acid in the millet bran and other cereal materials (*5*, *35*). Interestingly, reducing the particle size of the bran increased the mass fraction of gallic and *p*-coumaric acids in bread, but decreased mass fraction of 4-hydroxybenzoic and vanillic acids, while xylanase pretreatment increased the mass fraction of ferulic and *p*-coumaric acids. In addition, xylanase enhanced the mass fraction of gallic acid in the bread made with medium-sized bran. Fibre solubilisation can contribute to the release of hydroxycinnamic acids, especially ferulic acid (*36*), which are otherwise bound to the hemicellulose of the plant cell walls (*35*). Still, the changes in mass fractions of individual phenolic acids did not affect the total content of the phenolic acids, which remained statistically the same despite the bran treatments. However, there is a possibility that the antioxidant capacity of the bread was affected, since some studies have shown that in addition to the phenolic acid content, their composition and the degree of substitution of the xylan backbone influence the antioxidant activity of millet (*16*, *35*).

### Influence of bran particle size and xylanase on bread physical properties

All physical properties of bread except crumb springiness (data not shown) were significantly affected by the interaction of particle size and xylanase addition (p<0.02; Table 3 and Table 4). Bread made with coarse bran had the highest specific volume, but the difference between the bread samples was small (≤10%) (Table 4). The specific volume of the bread made with bran was similar to the control rice bread (*v*=1.5 mL/g) in our previous study (*5*), where the addition of fine (*d*_50_=26 µm) or coarse (*d*_50_=172 µm) millet bran resulted in much higher specific volume (*v*=2 mL/g) of the bread. Such difference could be due to not only a larger difference in the bran particle size in this study, but also to the difference in the chemical composition of the bran, namely its lower fat mass fraction. Similar to this study, Noort *et al*. (*15*) found that coarser wheat bran yielded the bread with a higher volume than fine bran, which is due to the reduction in the molecular mass of arabinoxylans by severe grinding (*17*). Here, xylanase pretreatment positively affected only the volume of the bread made with medium bran, possibly due to an increase in SDFS mass fraction (Table 2). Water-soluble arabinoxylans positively impact bread volume due to an increase in dough viscosity, stabilisation of gas bubbles, and their retention in the dough (*20*, *37*).

**Table 4:** Physical properties of gluten-free bread depending on the bran particle size (coarse, medium or superfine) with or without xylanase (X) addition

Parameter	Coarse	Coarse+X	Medium	Medium+X	Superfine	Superfine+X
*v*/(cm^3^/g)	(1.69±0.04)^a^	(1.62±0.03)^ab^	(1.51±0.06)^c^	(1.59±0.04)^b^	(1.56±0.05)^bc^	(1.56±0.06)^bc^
L*	(56.1±0.3)^b^	(55.2±0.4)^c^	(56.9±0.3)^a^	(54.6±0.4)^d^	(53.2±0.5)^e^	(51.2±0.5)^f^
a*	(5.38±0.09)^b^	(5.2±0.2)^b^	(5.3±0.1)^b^	(5.39±0.09)^b^	(6.1±0.3)^a^	(6.0±0.2)^a^
b*	(18.6±0.2)^d^	(17.9±0.3)^e^	(19.5±0.3)^c^	(19.4±0.2)^c^	(22.0±0.4)^a^	(20.9±0.2)^b^
Hardness/N	(26.6±0.6)^b^	(27.2±0.8)^b^	(31.2±0.9)^a^	(26.2±1.2)^b^	(25.5±1.0)^b^	(22.1±1.3)^c^
Resilience	(0.30±0.01)^bc^	(0.279±0.007)^c^	(0.283±0.008)^c^	(0.302±0.006)^b^	(0.32±0.01)^ab^	(0.32±0.02)^a^
Cohesiveness	(0.57±0.012^ab^	(0.54±0.01)^c^	(0.54±0.01)^c^	(0.552±0.003)^bc^	(0.57±0.02)^ab^	(0.59±0.02)^a^
Chewiness/g	(14.6±0.5)^b^	(14.2±0.7)^bc^	(16.4±0.7)^a^	(14.0±0.6)^bc^	(14.5±0.4)^bc^	(13.1±1.0)^c^
Maximum viscosity/BU	(211.5±4.9)^ab^	(201.0±8.5)^b^	(221.0±0.1)^a^	(176.5±2.1)^c^	(198.0±0.1)^b^	(200.5±3.5)^b^
Cold paste viscosity/BU	(429.5±7.8)^ab^	(397.0±19.8)^b^	(445.5±2.1)^a^	(330.0±8.5)^c^	(397.0±1.4)^b^	(395.5±7.8)^b^
Setback viscosity/BU	(216.0±2.8)^ab^	(194.0±11.3)^bc^	(223.0±2.8)^a^	(151.0±5.7)^d^	(196.5±0.7)^bc^	(192.5±5.0)^c^

The results are expressed as mean value±standard deviation, *N*≥2. *v*=specific volume, L*= lightness, a*=redness and b*=yellowness, BU=Brabender units. Mean values with different letters in superscript within the same row are significantly different at p<0.05 according to Tukey’s test

The crumb colour was similar in all bread samples; it was dark, with reddish (a*) and yellowish (b*) tones. The addition of bran gives the bread a dark colour (*5*, *38*, *39*). The crumb was the darkest and reddest in the bread with superfine bran. In agreement, Coda *et al*. (*16*) showed that smaller bran particle sizes (50 and 160 µm) provide darker, but more uniform bread colour. In this study, xylanase interacted with particle size to slightly reduce the lightness and yellowness of the bread. A possible explanation for the darker and redder colour is the fibre solubilisation, which may contribute to the Maillard reactions and caramelisation (*37*).

Differences in crumb texture among the bread samples were small (Table 4). Crumb chewiness was strongly correlated with hardness (R=0.94). Compared to our previous study in which bread enriched with millet bran had a softer crumb that was easier to chew (*5*), here, the crumb hardness and chewiness were closer to those of plain rice bread. This could be related to the lower specific volumes of bread measured in this study. Nevertheless, bread with medium sized bran pretreated with xylanase showed significantly lower crumb hardness and chewiness with improved resilience than the bread with untreated medium size bran. This could be related to the highest increase in SDFS mass fraction (Table 2). In agreement, other authors reported that xylanase softens gluten-free bread (*21*, *40*). A similar decrease of chewiness but also cohesiveness was noticed when xylanase was added to foxtail millet bread (*40*). In this work, with the use of xylanase, crumb cohesiveness dropped only in the bread with coarse bran. Coda *et al*. (*16*) found that the addition of bioprocessed wheat bran with a particle size of 160 µm resulted in the softest crumb, but also the highest specific volume, compared to bran with larger (750 and 450 µm) and lower (50 µm) particle sizes.

Gluten-free bread is known for its rapid staling (*38*). The maximum viscosity of bread can be related to polysaccharide composition and hydrolytic action of enzymes, while the cold paste viscosity and setback viscosity are indicators of the rate of starch retrogradation and bread shelf-life (*40*). The viscosity values of bread with medium bran pretreated with xylanase were the lowest among the samples (Table 4). The peak, cold paste and setback viscosity were inversely correlated with the amount of xylanase (R=-0.64, R=-0.69 and R=-0.71, respectively). In agreement, Leys *et al.* (*41*) established significantly lower viscosity of the wheat dough after fermentation and of the baked bread supplemented with xylanase. In contrast, Sarabhai *et al*. (*40*) reported an increase in hot paste, cold paste and setback viscosity of foxtail millet batter due to higher starch content and insignificant action of xylanase. Such discrepancy could be due to a much higher xylanase amount in their study, a different enzyme origin, and the fact that we measured the viscosity of bread. Furthermore, Lebesi and Tzia (*19*) reported that xylanase treatment retarded staling of wheat cakes enriched with oat and rice bran, which was attributed to higher water retention and fibre solubilisation, acting as hydrocolloid. Here, we can link the lowest viscosity of bread containing xylanase-pretreated medium-sized bran with the highest hydrolytic action of the enzyme on non-starch polysaccharides and a possibly slower staling rate of the bread during storage.

### Principal component analysis

**Fig. 1:**
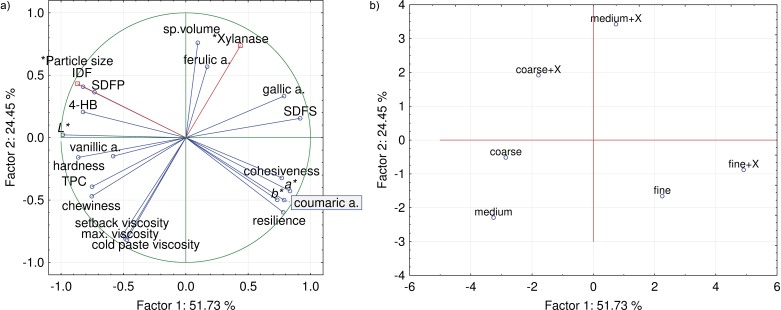
Factor scores and loadings plot from principal component analysis of physical parameters and bioactives of gluten-free bread samples with millet bran of different particle size (coarse, medium or fine) and xylanase addition (X). a.=acid, 4HB=4-hydroxybenzoic acid, IDF=insoluble dietary fibre, L*=lightness, a*=redness, b*=yellowness, SDFP=fibre soluble in water but precipitated in 78% aqueous ethanol, SDFS=fibre soluble in water and not precipitated in 78% aqueous ethanol, Sp.=specific; TPC=total phenolic content

The PCA of physical properties, fibre and phenolic content of gluten-free bread as a function of bran particle size and xylanase treatment extracted five factors. The first two factors with eigenvalues of 10.3 and 4.9 accounted for 76% of the total variation (Fig. 1). The first component of the variables (Fig. 1a) contrasts crumb redness, yellowness, resilience, cohesiveness, SDFS mass fraction, coumaric and gallic acids with crumb lightness, hardness, chewiness, the mass fraction of SDFP, IDF, TPC, 4-hydroxybenzoic and vanillic acids, and is related to bran particle size. Consequently, the projection of the cases (Fig. 1b) along component 1 essentially separates the loaves of bread with coarse and medium-sized bran from the samples with superfine bran. The second component contrasts bread specific volume and ferulic acid mass fraction with viscosities, which was related to the presence of xylanase. Thus, the sample with medium sized bran pretreated with xylanase was characterised by high specific volume and high mass fraction of ferulic acid. On the contrary, bread with medium, coarse or fine size bran without xylanase was related to high viscosities, crumb chewiness and hardness, as well as high TPC. Bread made with superfine bran resembled the bread made with coarse and medium size bran without xylanase in terms of viscosities. As the xylanase pretreatment gave the best results with medium-sized bran, the medium-sized bran with or without xylanase pretreatment was selected for further nutritional and sensory evaluation.

### Nutritive value, protein digestibility and mineral bioacessibility of bread

The composition and nutritional value of the bread made with medium-sized bran did not differ significantly as a function of xylanase pretreatment (Table 5). As expected, all gluten-free bread samples had a high mass fraction of carbohydrates, but low fat mass fraction. Still, the carbohydrate mass fraction was 7.5% lower in the bread containing bran than the rice bread, which was due to approx. 70% higher dietary fibre content, because the control bread was previously shown to have low fibre mass fraction (2.5 g/100 g) (*5*). Li *et al.* (*10*) showed that the addition of 2% millet fibre slowed the starch digestibility of steamed bread, so that the estimated glycaemic index decreased from high to medium. A synergy between millet fibre and phenolic compounds, as well as the interaction of starch with its proteins and lipids, are beneficial in controlling blood glucose levels (*9*).

**Table 5:** Nutrient composition, *in vitro* protein digestibility and mineral bioaccessibility of gluten-free bread with medium-sized bran (averaged values of xylanase treated and untreated) compared to control rice bread

Parameter	Control rice bread	Bread with medium-sized bran	Bread with medium-sized bran and xylanase
Energy/kJ and (kcal)	898 (225)	869 (217)	878 (219)
*w*(fat)/(g/100 g)	1.2±0.1	1.6±0.1	1.6±0.1
*w*(carbohydrate)/(g/100 g)	46.8	42.9	43.3
of which *w*(sugar)/(g/100 g)	2.0±0.1	2.0±0.1	2.1±0.1
*w*(protein)/(g/100 g)	5.5±0.2	5.5±0.1	5.5±0.1
*w*(protein digestibility)/%	87±1	85±1	84±1
*w*(total mineral as ash)/(g/100 g)	1.8 ± 0.8	2.0±0.1	1.9±0.1
*w*(Mg)/(mg/100 g)	8.0±0.4	12.8±0.1	12.8±0.2
*w*(Mg)_bioaccessible_/%	68	67	64
*w*(Zn)/(mg/100 g)	0.61±0.03	0.62±0.01	0.61±0.01
*w*(Zn)_bioaccessible_/%	36	35	45
*w*(Fe)/(mg/100 g)	0.13±0.02	0.56±0.01	0.53±0.01
*w*(Fe)_bioaccessible_/%	23	18	24
*w*(Cu)/(mg/100 g)	0.14±0.01	0.23±0.01	0.20±0.01
*w*(Cu)_bioaccessible_/%	33	48	83

The results are expressed as mean value±standard deviation, *N≥*2

Cereal proteins are known to be less digestible than animal proteins (*42*). The protein content of the bread did not change with the addition of the millet bran, but their digestibility was slightly impaired (Table 5), regardless of the xylanase pretreatment. Similarly, Li *et al*. (*10*) showed that the protein digestibility of steamed bread containing millet flour and bran decreased with increasing fibre mass fraction. Yet, protein digestibility of millet is most negatively affected by polyphenols (*9*). We therefore assume that the impairment of protein digestibility of the bread with millet bran was affected by the increase in fibre and phenolic mass fraction.

After a 10% replacement of rice flour with millet bran, the bread was enriched with total minerals (15%), including iron (341%), magnesium (74%), copper (56%) and zinc (7.5%) (Table 5), irrespective of xylanase pretreatment. After adding untreated bran to the bread, the bioaccessibility of magnesium and zinc was unchanged, but it was lower for iron and higher for copper than in the control bread. Xylanase pretreatment of bran improved the bioaccessibility of zinc and copper compared to the other two bread samples. In addition, xylanase pretreatment improved the bioaccessibility of iron from the enriched bread compared to bread with untreated bran. Overall, bioaccessibility of minerals is negatively affected by insoluble fibre, and positively by soluble fibre (*43*). Xylan readily forms complexes with Fe^2+^ and Zn^2+^, and thus reduces their uptake during absorption (*11*). In agreement, Ramonaltyté *et al.* (*44*) also reported increased bioaccessibility of zinc in bread after adding xylanases, whereas Baye *et al.* (*11*) reported an increase in the bioaccessibility of iron in wheat flour, sorghum flour and teff upon xylanase treatment. We assume that the bioaccessibility of minerals was favoured by the higher mass fraction of free phenolics and soluble fibre after the xylanase treatment. Moreover, Krishnan *et al.* (*45*) showed that the bioaccessibility of iron from finger millet seed coat is negatively affected by phytic acid content, while the bioaccessibility of zinc is obstructed by polyphenols. Soaking the bran before baking reduces phytate content through the action of endogenous phytase (*43*). The millet bran from this study contained *w*(phytate)=1.22%, which were reduced to 0.59% after soaking without the enzyme addition, and to 0.56% in the presence of xylanase. Similar mass fractions on dry mass basis of phytic acid (1.0−1.3%) were previously found in finger and pearl millet bran (*43*, *45*). Here, we found that the addition of millet bran after xylanase pretreatment can enrich gluten-free bread with iron while maintaining the same level of bioaccessibility.

### Sensory attributes of bread

**Fig. 2:**
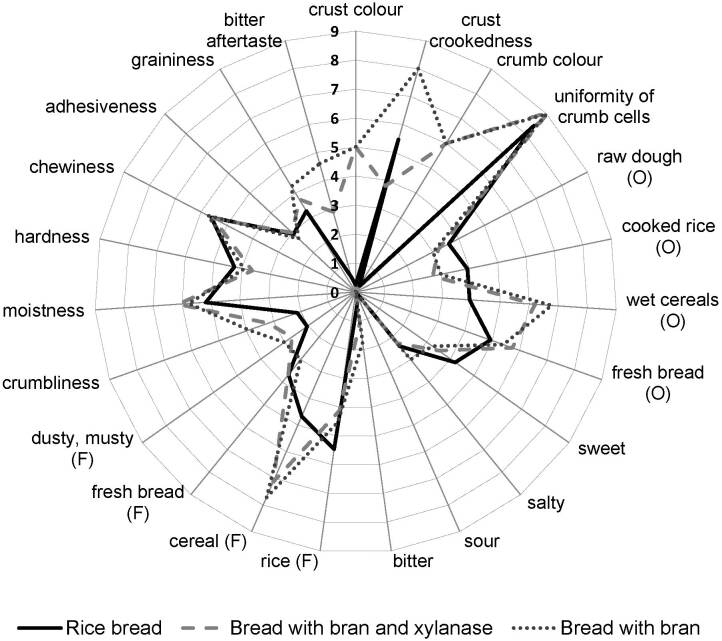
Spider diagram of descriptive sensory analysis of gluten-free bread with medium-sized millet bran with or without xylanase compared with control rice bread (*N*=16 participants). O=odour, F=flavour

**Fig. 3:**
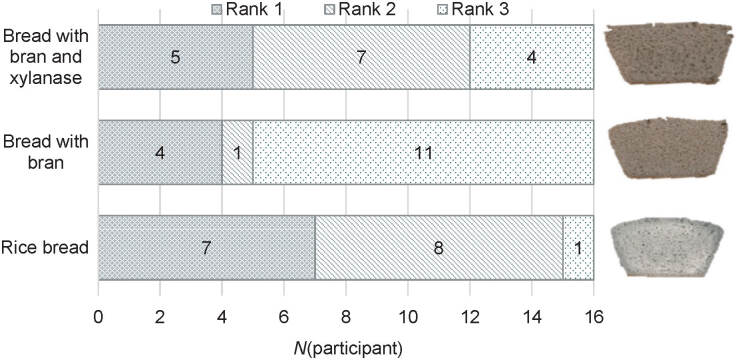
Distribution of ranks in preference test of pan gluten-free bread with medium-sized millet bran with or without xylanase compared with control rice bread (*N*=16 participants)

Cereal by-products in excessive amounts can negatively affect the sensory properties of baked products (*46*). Fig. 2 and Fig. 3 show the results of descriptive analysis and preference ranking of bread, respectively. Bran addition increased crust and crumb darkness compared to the control rice bread, as demonstrated by the instrumental measurements as well as in previous studies (*39*, *47*). A darker colour is desirable since gluten-free bread usually has lighter colour than wheat bread (*48*). Bran addition also increased wet cereal odour, cereal flavour, bitter taste and aftertaste. The undesirable bitterness can be attributed to phenolic compounds, residues of phytic acids or phytates, amino acids and peptide content (*49*). It also affected bread structure by increasing its crust crookedness, crumbliness, graininess and moistness. Higher crumbliness and graininess are expected in bran- and fibre-enriched bread (*50*, *51*). Foste *et al.* (*39*) explained the juicier texture (which might be correlated with moistness) of gluten-free bread with quinoa bran with the higher water binding capacity due to the higher protein and fibre content.

Xylanase pretreatment of millet bran reduced its negative effects on the sensory properties of the bread. Bread with the addition of xylanase-treated millet bran was characterised by lower crust crookedness, graininess, as well as lower intensity of bitter taste and aftertaste than the bread with untreated millet bran. Ghoshal *et al*. (*52*) also reported a smoother texture of wholemeal wheat bread when prepared with xylanase. Contrary, according to Nikinmaa *et al.* (*53*), xylanase had no effect on the bitterness of wholegrain crackers. Similarly to this study, biscuits containing millet bran and xylanase have been found to have a low bitter aftertaste (*18*).

All bread samples were similarly moderately liked, with mean hedonic scores for rice bread 6.9, bread with bran 6.5, and bread with bran and xylanase 6.9. Friedman’s ANOVA and Kendall’s coefficient of concordance, which was 0.168, indicated small differences at p=0.068 in the preference rankings of the bread samples. Still, the bread with added millet bran and xylanase was the most preferred (mean rank=1.6±0.6, sum 26), followed by the control bread (mean rank=1.9±0.8, sum 31) and finally the bran-enriched bread without xylanase (mean rank=2.4±0.9, sum 39). Similarly, 10% addition of rice bran was found to improve the overall sensory properties of gluten-free rice bread, as reported by Phimolsiripol *et al. (**38*).

## CONCLUSIONS

This study investigated the effect of adding millet bran of different particle sizes, with or without xylanase pretreatment on the physical, sensory and nutritional properties of gluten-free bread. The results showed that the grinding process and the resulting particle size of millet bran affect the physical properties as well as the dietary fibre, phenolic content and composition of gluten-free bread. Moreover, the effect of xylanase varies according to the particle size and is most efficient in the bran of medium particle size, which is similar to the granulation of conventional flour. Therefore, before it is used for baking, the treatment of millet bran with xylanase after ultracentrifugal grinding is more beneficial than after the several steps of cryogrinding. Replacing 10% of the rice flour with millet bran of medium particle size pretreated with xylanase substantially enriches the bread with minerals, especially magnesium, iron and copper. Millet bran addition does not affect the sensory liking of the bread, which showed to be moderate as that of plain rice bread. Xylanase affects the solubilization of dietary fibre and the release of phenolics, so it can be used to improve the bioaccessibility of zinc and copper from bread. It also promotes crust and crumb smoothness. Future studies should investigate the use of other non-thermal processes that could further enhance xylanase activity in millet bran.
